# Impact of eggshell membrane on metabolism and cell adhesion in oxidatively stressed canine chondrocytes

**DOI:** 10.3389/fvets.2024.1517349

**Published:** 2025-01-08

**Authors:** Juraj Vozar, Nikola Hudakova, Natalia Nosalova, Mykhailo Huniadi, Dana Marcincakova, Slavomir Hornak, Lubica Hornakova, Petra Majerova, Dasa Cizkova

**Affiliations:** ^1^Centre of Experimental and Clinical Regenerative Medicine, Small Animal Clinic, University of Veterinary Medicine and Pharmacy in Košice, Košice, Slovakia; ^2^Department of Pharmacology and Toxicology, University of Veterinary Medicine and Pharmacy in Košice, Košice, Slovakia; ^3^Small Animal Clinic, University of Veterinary Medicine and Pharmacy in Košice, Košice, Slovakia; ^4^Institute of Neuroimmunology, Slovak Academy of Sciences, Bratislava, Slovakia

**Keywords:** eggshell membrane, canine chondrocytes, oxidative stress, osteoarthritis, prevention, treatment

## Abstract

Eggshell membrane (ESM) is a rich source of bioactive compounds, including proteins, peptides, and antioxidants, contributing to its potential therapeutic benefits. These natural antioxidants might help neutralize reactive oxygen species (ROS) and modulate inflammatory responses, which are often linked with chondrocyte damage in osteoarthritis. In this study, we investigated the functional effects of ESM proteins on H_2_O_2_-induced oxidative stress in a neonatal canine chondrocytes. The isolated neonatal chondrocytes demonstrated a high proliferation rate and increased glycosaminoglycan (GAG) production during cultivation. In addition, the expression of key cartilage markers, including collagen types II and IX, and aggrecan, confirmed the retention of the chondrocyte phenotype. Under *in vitro* conditions, post-treatment with ESM improved chondrocyte viability, indicating that ESM may have a reparative role in mitigating oxidative damage. This significant therapeutic potential was validated through XTT assays, which measured cell metabolic activity at 24 h, and Real-time Cell Analysis (RTCA), providing continuous monitoring over 98 h. In contrast, the preventive effects of ESM against stress were observed exclusively in the XTT analysis. By investigating these aspects, we provide insight into the potential of ESM proteins to protect chondrocytes from oxidative damage, particularly in cartilage repair and joint health. This study is one of the first to create a vital platform based on canine neonatal chondrocytes for monitoring dietary supplements designed to prevent or repair dog cartilage damage. Thus, the study offers a valuable contribution to understanding how ESM bioactive compounds can be used therapeutically, bridging the gap between *in vitro* findings and practical applications in veterinary medicine.

## 1 Introduction

Eggshell membrane, a natural byproduct of the egg industry, has gained significant attention as a valuable source of bioactive compounds with therapeutic potential. The key proteins in ESM, including collagens, hyaluronic acid, and chondroitin sulfate play a fundamental role in cartilage health (1). While collagens provide tensile strength to the extracellular matrix (ECM) of cartilage, glycosaminoglycans (GAGs) such as chondroitin sulfate and hyaluronic acid (HA) help maintain hydration and elasticity (2). Furthermore, collagen types I, V, and X, elastin, and various growth factors included in ESM are critical for supporting cartilage repair (3). These properties make ESM an attractive candidate for promoting cartilage repair, especially in degenerative conditions like osteoarthritis (4). Similar to humans, dogs experience osteoarthritis, where chronic inflammation triggers oxidative stress, causing chondrocyte death, cartilage breakdown, and degradation of the ECM, ultimately leading to joint degeneration (5). ESM has been shown to decrease pro-inflammatory cytokines and improve joint health in animal models and human studies, making it a popular ingredient in dietary supplements (6). However, there is a significant gap in the literature regarding the role of ESM in combating oxidative stress, a key factor in cartilage degeneration and chondrocyte apoptosis. Oxidative stress results from an imbalance between reactive oxygen species (ROS) and the body’s antioxidant defenses, leading to cellular damage and joint dysfunction (7). ESM contains natural antioxidants, such as lysozyme and ovotransferrin, which can neutralize ROS, thereby reducing oxidative stress (8, 9). Furthermore, the antioxidative effects of ESM hydrolysates were evidenced by a significant increase in glutathione levels in H₂O₂-treated Caco-2 cells. Glutathione is a critical antioxidant that directly neutralizes ROS, and regenerates other antioxidants, such as vitamins C and E to their active forms (10). By modulating the immune response and lowering ROS levels, ESM helps protect chondrocytes, particularly in conditions where oxidative damage accelerates cartilage degradation (11). Exploring this aspect is essential for gaining a more comprehensive understanding of ESM’s potential in cartilage repair, while also advancing the development of optimized *in vitro* chondrogenic models to test these dietary supplements effectively (12). Traditionally, studies on chondroprotection have focused on adult or osteoarthritic (OA) chondrocyte models because these systems reflect the degenerative changes observed in osteoarthritis (13). However, recent advances have highlighted the advantages of using juvenile (cells derived from young tissue) or neonatal chondrocyte (cells derived from newborn tissue) models for studying cartilage repair and regeneration due to their distinct higher proliferation rate and synthesis compared to adult chondrocytes (14). Juvenile and neonatal chondrocytes preserve their chondrogenic potential for extended periods, even in 2D cultures (15). They continue to produce key cartilage-specific proteins, including collagen type II (COL II), aggrecan, and GAGs, which are essential for maintaining a stable chondrocyte phenotype *in vitro* studies (16, 17). Building on these findings, we have developed an optimized canine neonatal chondrocyte model that offers a valuable *in vitro* platform for investigating cartilage prevention and repair strategies in veterinary regenerative medicine. This is the first study to investigate whether ESM proteins can protect canine neonatal chondrocytes from damage caused by oxidative stress. Exploring the preventive and therapeutic potential of ESM may uncover novel therapeutic strategies to enhance chondrocyte survival and preserve cartilage function, offering valuable insights for veterinary practice.

## 2 Materials and methods

### 2.1 Establishment of canine neonatal chondrocyte culture

#### 2.1.1 Animals and tissue sampling

Four full-term puppies that died due to hypoxia during delivery (cesarean section) were used in our study. Their body weight was normal ranging between 410 and 550 g concerning the breed (German Shepherd) and the age at the time of death. Cadavers were utilized for tissue dissection following the acquisition of informed consent signed by the owners. The procedure was conducted with the approval of the Ethics Committee of the UVLF Kosice (EKVP/2022-21). The cartilage tissue was harvested from the proximal and distal epiphyses of the humerus (shoulder and elbow joint) and femur (hip and knee joint) (*n* = 24). In addition, cadaveric specimens were utilized also for harvesting skin tissue samples (2 × 2 cm) from the abdominal region (*n* = 4). These samples were subsequently used in a fibroblast isolation procedure. The dissection procedure was performed under sterile conditions using a Biological Safety Cabinet (Herasafe, Class II Biological Safety Cabinet, Thermo Fisher Scientific Inc., United States, BioSafe, Class II. A2, Ekokrok, SK), preventing unwanted pathogens from contaminating cartilage and fibroblast samples.

#### 2.1.2 Primary cultures of neonatal chondrocytes

The pieces of dissected articular cartilage were washed extensively in Hank’s Balanced Salt Solution (HBSS, Sigma, CA, United States) with a high concentration of antibiotic/antimycotic solution (hATBM), containing 300 U/ mL penicillin, 300 μg/mL streptomycin, 5 μg/mL amphotericin B and 15 μg/mL gentamycin (Lonza, Switzerland). Subsequently, cartilage fragments were digested in 1 mg/mL collagenase type I and type IV (Gibco, Invitrogen, Carlsbad, CA, United States) in Dulbecco’s phosphate buffer saline (DPBS, Gibco, United States) supplemented with hATBM for 2–4 h at 37°C. After digestion, the collagenase was neutralized with the same volume of DPBS containing 10% fetal bovine serum (FBS) (Sigma, United States), and 2% penicillin/streptomycin (Gibco, United States). Afterwards, the cell suspension was filtered through a 70 μm cell strainer and centrifuged at 300 × *g*/10 min. The pellet was re-suspended in 1 mL of Dulbecco’s modified Eagle’s medium High Glucose (DMEM, Sigma, CA, United States), and cells were counted using a hemocytometer and a 4% trypan blue solution.

#### 2.1.3 Morphology, passaging, and cryopreservation

Neonatal chondrocytes (nChoN) were suspended in DMEM supplemented with 10% FBS (Sigma, United States), 1% antibiotic-antimycotic [ATB/ATM, penicillin (100 U/mL), streptomycin (100 μg/mL), amphotericin B (2.5 μg/mL) and gentamicin (5 μg/mL) (Lonza, Switzerland)], plated in the concentration of 0.5 × 10^6^ on a 25 cm^2^ tissue culture flask/T25 cm^2^ (Corning, Lowell, MA, United States, seeding density 0.2 × 10^4^ cells/cm^2^) and incubated at 37°C and 5% CO_2._ The first medium change was after 24 h, and the following medium changes were every 2–3 days. The morphology of primary nChoN cultures at 24 h, 48 h, and 72 h was evaluated using the inverted microscope Zeiss Axiovert 200 equipped with a digital acquisition system. Primary chondrocytes at 80–90% confluence were subcultured to obtain passage 1 (P1) by washing with DPBS and released from each T25 culture flask using 1 mL of 0.05% trypsin/EDTA solution (Sigma, CA, United States), and incubated at 37°C for 3–7 min. Trypsin was neutralized with the same volume of working culture medium DMEM containing 10% FBS, and 1% penicillin/streptomycin (Gibco, United States), the cell suspension was centrifuged at 300 × *g*/10 min and pellets (*n* = 2) were re-suspended in DMEM, and cells were counted and subsequently plated for further analyses. The remaining pellets (*n* = 2) were suspended in freezing media with 50% DMEM supplemented with 40% FBS and 10% dimethyl sulfoxide (DMSO, Sigma, United States), frozen at 1°C/min (MrFrosty Freezing Container, Sigma, United States) in −80°C for 24 h and subsequently stored in liquid nitrogen.

#### 2.1.4 Chondrocyte growth curve assessment

The growth curve of cultured nChoN was evaluated using trypan blue exclusion. Chondrocytes were seeded in 24-well polystyrene culture plates (TPP^®^, Switzerland) at a concentration of 10 × 10^3^ cells per well in 500 μL of DMEM supplemented with 1% ATB/ATM and 10% FBS. The cells were cultured for up to 4 days. Trypan blue staining was performed daily to assess cell viability and the total number of cells.

#### 2.1.5 Isolation and cultivation of dermis-derived fibroblasts

Skin samples were washed in 70% ethanol for 10 s, followed by sterile 1 × DPBS (pH 7.4) for 15 s. The dermis and epidermis were then separated by enzymatic digestion using Dispase II (1.0 U/mL in DMEM) supplemented with 1% ATB/ATM at 37°C for 2 h. A digested epidermal fraction was centrifuged at 300 × *g* for 10 min, cryopreserved and stored in a cryobank, while the undigested dermis was processed further. The dermal tissue was cut into small fragments using sterile scissors, washed in sterile DPBS (pH 7.4), transferred to a 15 mL tube containing 1 mL of collagenase type I (0.5 mg/mL) in DPBS and incubated at 37°C for 10–12 h. The cell suspension was centrifuged at 300 × *g* for 10, the cell pellet was resuspended in DMEM supplemented with 10% FBS, 1% ATB/ATM. The cells were then seeded at a density of 0.5 × 10^6^ cells into T25 cm^2^ flasks in a humidified incubator at 37°C with 5% CO_2_. When the cells reached 90–100% confluence, they were either passaged or harvested for further experiments.

#### 2.1.6 Alcian blue staining for glycosaminoglycans

Alcian blue staining was performed to detect GAGs in the ECM of nChoN at passage 1 (P1). Chondrocytes were seeded in 24-well culture plates at 2500 cells/cm^2^ and cultivated as a monolayer. Fibroblasts, cultured under the same conditions, were used as a negative control. After 1 week of cultivation in DMEM supplemented with 10% FBS and 1% ATB/ATM, the medium was removed. The chondrocytes and fibroblasts were washed with DPBS and fixed with 4% formaldehyde (Sigma-Aldrich, United States) for 10 min. To stain the GAGs, a 1% alcian blue solution (Sigma-Aldrich, United States), prepared in distilled water, was added to the culture plates and incubated for 30 min. Following incubation, the staining solution was removed, and the cells were washed first with 0.1 N hydrochloric acid (HCl, Sigma-Aldrich, United States) and then with distilled water. The staining procedure was completed by applying a mounting medium (Entellan, Sigma-Aldrich, United States) to the cell culture, followed by placing a cover glass over the sample. The stained ECM of nChoN and fibroblasts was observed under a light inverted microscope Zeiss Axiovert 200 (Zeiss, Germany) equipped with a digital acquisition system.

#### 2.1.7 Immunocytochemistry of chondrocytes

Neonatal chondrocytes (P1) were seeded onto Lab-Tek chamber slides (Nunc^®^ Lab-Tek^®^ Chamber Slide™ system, Thermo Fisher Scientific, United Kingdom) at a density of 1 × 10^4^ cells per well in monolayer culture. After reaching confluency (1 week), cells were fixed with 4% paraformaldehyde (PFA) in DPBS for 10–15 min at room temperature to preserve the cellular architecture and proteins. To reduce non-specific binding and improve permeabilization, cells were incubated with 10% normal goat serum (NGS) in PBS containing 0.1% Triton X-100 (PBS TX100) for 60 min at room temperature. Primary antibodies were diluted in 1% NGS in PBS TX100 as follows: Collagen Type II: Polyclonal rabbit Anti-Collagen II antibody (ab34712, dilution 1: 100, Abcam, United States). Collagen IX: Polyclonal rabbit Anti-Collagen IX antibody (ARG55560, dilution 1: 200, Origo Laboratories, United States). Aggrecan: Monoclonal mouse Aggrecan antibody (BC-3 clone, dilution 1: 500, Invitrogen, MA3-16888, United States). Cells were incubated with the primary antibody mixture overnight at 4°C. The next day, cells were washed 3 times with DPBS for 5 min each to remove unbound primary antibodies. Cells were incubated with fluorophore-conjugated secondary antibodies diluted in blocking solution for 1 h at room temperature in the dark: Anti-rabbit secondary antibody: Texas Red-conjugated (Alexa Fluor 594, at 1:250 dilution), Anti-mouse secondary antibody: FITC-conjugated (Alexa Fluor 488, dilution 1:250). DAPI (1,250) was added during this step to counterstain the nuclei. Next, cells were washed 3 times with DPBS for 5 min each to remove excess secondary antibodies and DAPI. Negative controls were performed by omitting the primary antibody for each chondrogenic marker, while incubation with corresponding secondary antibodies, was followed as per the standard protocol. This approach ensures that observed staining is solely attributed to the unspecific secondary antibody binding. Subsequently, Vectashield mounting medium (Abcam, United States) was applied to the slides to preserve the fluorescence signal and prevent any loss of intensity. Finally, the slides were coverslipped. Representative images were captured using a Zeiss Axio Observer Z1 microscope equipped with an Apotome 3 fluorescence module. The following objectives were utilized to ensure excellent resolution: EC Plan-Neofluar (10x/0.3 Ph1) and LD Plan-Neofluar (40x/0.6 Ph2 Korr., Zeiss, Germany). The presence of collagen type II, collagen IX, and aggrecan was confirmed by visualizing distinct fluorescence signals corresponding to each protein using a multichannel fluorescence filter cube FS81 (DAPI-FITC-Rhodamine-Cy5) and COLIBRI 7 illumination systems.

### 2.2 Eggshell membrane

Eggshell membrane ESM^®^ (Torolis Explotaciones S.L, Spain) is a naturally derived ingredient obtained from the inner membrane of eggshells produced through mechanical action, washing, and crushing. According to the product description, eggshell membrane (ESM^®^) contains approximately 35% native collagen (Type I, V, and X) and about 6% GAGs, including chondroitin sulfate, hyaluronic acid and keratan and dermatan sulfates. It also contains an appreciable amount of lysozyme, about 3.5%. The natural extraction process ensures that ESM^®^ remains a 100% natural ingredient, free from synthetic additives, making it suitable for natural and sustainable products.

#### 2.2.1 Proteins extraction from ESM

For the extraction of proteins bound to the ESM, we have included Triton X-100 at 0.5 g or 1.0 g in 100 mL of 50 mM Tris–HCl buffer pH 8.8 according to a previously published protocol (18). A dissolving buffer (1,000 μL) was added to 100 mg of ESM powder in a 1.5 mL centrifuge tube. The solution was vortexed for 60 s and then allowed to dissolve at 4°C overnight. The ESM solution was centrifuged two times for 10 s each at 4000 × *g* to remove the insoluble materials. The supernatant (400 μL) was collected and 800 μL of acetone was added and left at 4°C overnight to precipitate the ESM proteins. The next day, the mixture was centrifuged at 10,000 × *g* for 40 min at 4°C. The supernatant was discarded, and the protein pellet was resuspended in 30 μL nano-water. The amount of extracted proteins from detergent was measured by NanoDrop (NanoDrop 2000c Spectrophotometer, Thermo Fisher Scientific/Marshal Scientific, United States). Aliquots (30 μL each) were stored at −80°C. The amount of extracted eggshell proteins from detergent solutions was measured using a NanoDrop by assessing absorbance at 280 nm due to the presence of aromatic amino acids like tryptophan and tyrosine. This method allows for quick and accurate determination of protein concentration in extraction solutions, making it suitable for assessing the efficiency of protein extraction from eggshells in the detergent solutions. The protein concentrations of four samples, measuring 42.34 mg/mL, 47.99 mg/mL, 59.90 mg/mL, and 62.32 mg/mL, were considered appropriate for *in vitro* chondrocytes experiments based on a previous study (16).

### 2.3 XTT assay: chondrocyte metabolic activity after exposure to ESM and H₂O₂

First-passage (P1) chondrocytes were seeded into two 96-well culture plates (Corning, Gibco, United States) at a density of 10 × 10^3^ cells per well in 100 μL of DMEM supplemented with 1% ATB/ATM and 5% FBS. The cells were allowed to adhere and grow for 24 h. In the first plate, the cells were exposed to ESM dissolved in DMEM at concentrations of 0.5, 1.0, and 1.5 mg/mL for 24 h. The second plate was used to evaluate the cells’ response to oxidative stress induced by H_2_O_2._ After a 24 h incubation in DMEM, the cells were incubated in DMEM (control) or exposed to H_2_O_2_ at concentrations of 100 μM (H100) and 200 μM (H200) for 60 min. The XTT assay was then performed in triplicates (*n* = 3) for each condition. A fresh XTT solution was prepared by mixing 5 mL of XTT labeling reagent with 0.1 mL of electron coupling reagent. Following this, 50 μL of the XTT mixture was added to each well, and the plates were incubated for 4 h at 37°C with 5% CO₂. Absorbance was measured at 492 nm using the APOLLO Absorbance Reader (Berthold Systems, United States) to determine cell metabolic activity.

### 2.4 XTT assay: cytoprotective effect of ESM on stressed chondrocytes

The impact of ESM pre-treatment: chondrocytes were seeded into 96-well plates at a density of 10 × 10^3^ cells per well and cultured in the presence of ESM at concentrations of 0.5, 1.0, and 1.5 mg/mL for 24 h. Following this incubation, the cells were exposed to H200 for 60 min to induce oxidative stress. After the H₂O₂ treatment, the cells were washed with DPBS to remove any residual H₂O₂ and incubated for 24 h. The pre-treatment effect of ESM on the H_2_O_2_-damaged-stressed chondrocytes was then evaluated using an XTT assay (identical procedure as described in 2.3).

The impact of ESM post-treatment: chondrocytes were seeded into 96-well plates at a density of 10 × 10^3^ cells per well and exposed to H200 for 60 min to induce oxidative stress. Following H₂O₂ treatment, the cells were washed with DPBS to remove residual H_2_O_2_. The cells were then incubated with ESM at concentrations of 0.5, 1.0, and 1.5 mg/mL for 24 h and DMEM for control. The post-treatment effect of ESM on the H_2_O_2_-damaged chondrocytes was subsequently assessed using an XTT assay (identical procedure as described in 2.3).

### 2.5 Real-time cell analysis

To further investigate the effects of ESM pre- and post-treatment on adherence and proliferation of H200-stressed chondrocytes for 3–4 days, the Real-Time Cell Analyzer (RTCA, Roche Applied Sciences, Mannheim, Germany) platform was employed. This technology uses electronic impedance detection to measure changes in a sample’s electrical properties, such as conductivity, which are influenced by cell membrane contact with the electrode and the strength of cell adhesion. These factors alter the electrical impedance of the golden electrodes on the surface of the E-plate (19). Chondrocytes (20 × 10^3^ cells in 150 μL medium per well) were seeded into 16-well E-plates (Roche, Mannheim, Germany) and cultured under the same ESM pre- and post-treatment conditions as those used for the XTT assay (identical procedure as described in 2.4). The experimental procedure followed the manufacturer’s instructions for the xCELLigence RTCA. The experiment was conducted over a total period of 98 h. Chondrocytes cultured in DMEM with 5% FBS and 1% ATB/ATM served as the control, while treatment with H200 alone was used to model oxidative stress. The cell index was normalized to a baseline value of 1 at 21 h of cultivation.

### 2.6 Statistical analysis

For each experiment, chondrocytes were pooled from four animals (*n* = 4), and the experiments were conducted in triplicate. The normality of the data was assessed using the Shapiro–Wilk test. Data from the XTT assay met the normality criteria and were analyzed using one-way ANOVA followed by Dunnett’s multiple comparisons test. In contrast, RTCA data did not meet the normality assumption and were analyzed using the Kruskal-Wallis test. Statistical information has been included, with values expressed as percentages relative to the control group, presented in Supplementary Table S1. All statistical analyses were performed using GraphPad Prism (version 10.3.1) for Windows, GraphPad Software, San Diego, CA, United States.

## 3 Results

### 3.1 Morphology of primary chondrocytes

**Figure 1:**
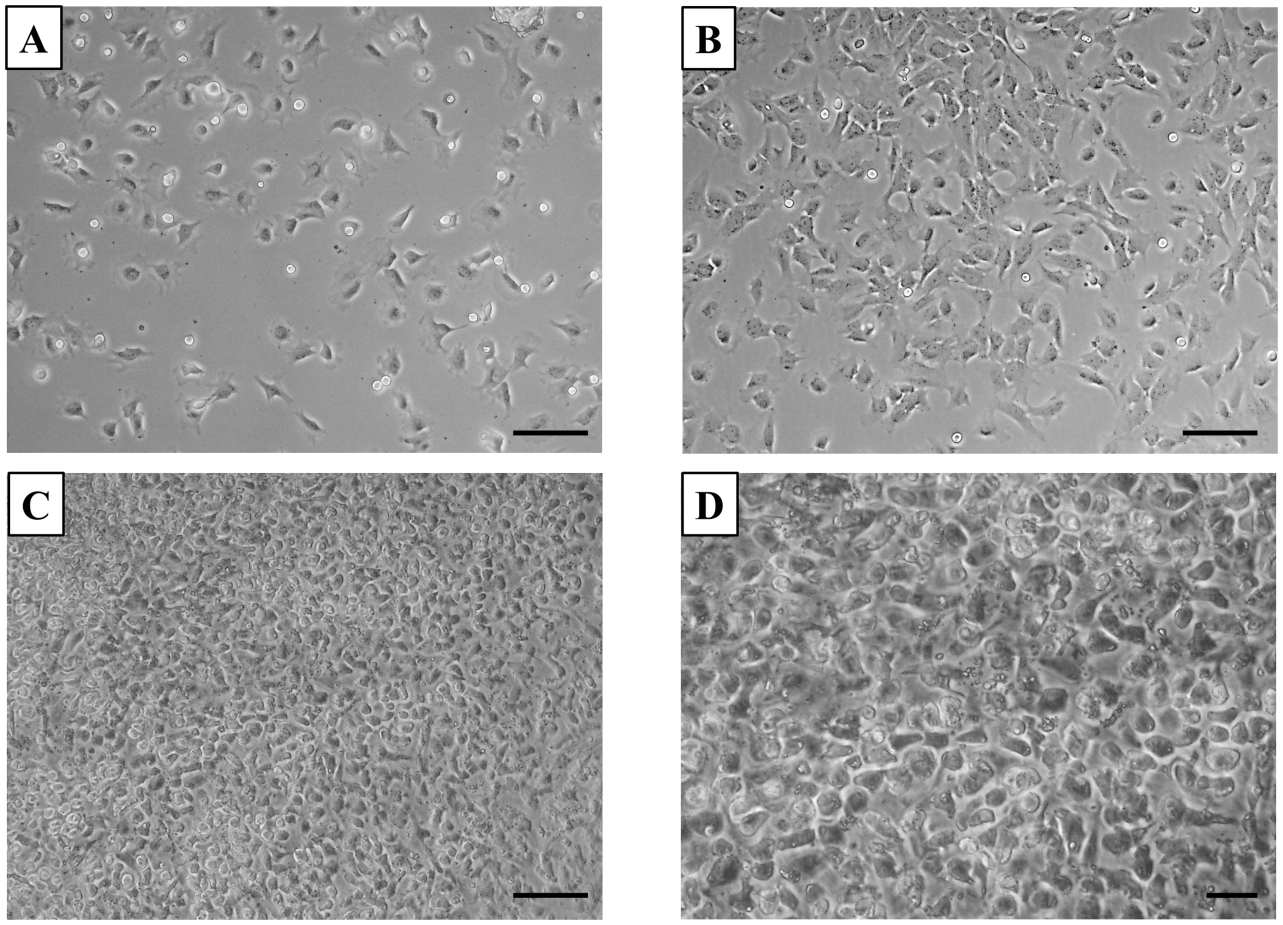
Primary culture of chondrocytes. **(A)** After 24 h of plating, most cells adhered to the surface, displaying a predominantly polygonal shape, with some light oval-shaped precursor cells revealing active division. **(B)** By 48 h, most cells adhered and started to adopt a fibroblast-like morphology. **(C,D)** At 72 h, the culture reached 100% confluency, with cells exhibiting increased spreading and a mix of polygonal and bipolar shapes. Scale bars: **(A–C)** = 50 μm, **(D)** = 20 μm.

In the primary chondrocyte culture prepared from neonatal canine articular cartilage, the morphological changes observed at different time points were typical for cells adapting to *in vitro* culture conditions. At 24 h post-plating, most cells were adherent, showing primarily a polygonal shape, with a few light oval precursor cells actively dividing (Figure 1A). By 48 h, polygonal cell numbers had increased, and chondrocytes began extending their processes, taking on a fibroblast-like shape, while decreasing light spherical precursor cells (Figure 1B). At 72 h, the neonatal chondrocytes developed a mixed morphology, displaying both polygonal and bipolar shapes, which coincided with increased expansion and spreading (Figures 1C,D). Such morphological changes are commonly observed in chondrocyte cultures as they adjust and spread, particularly when grown in monolayers.

#### 3.1.1 Chondrocyte growth curve assessment

**Figure 2:**
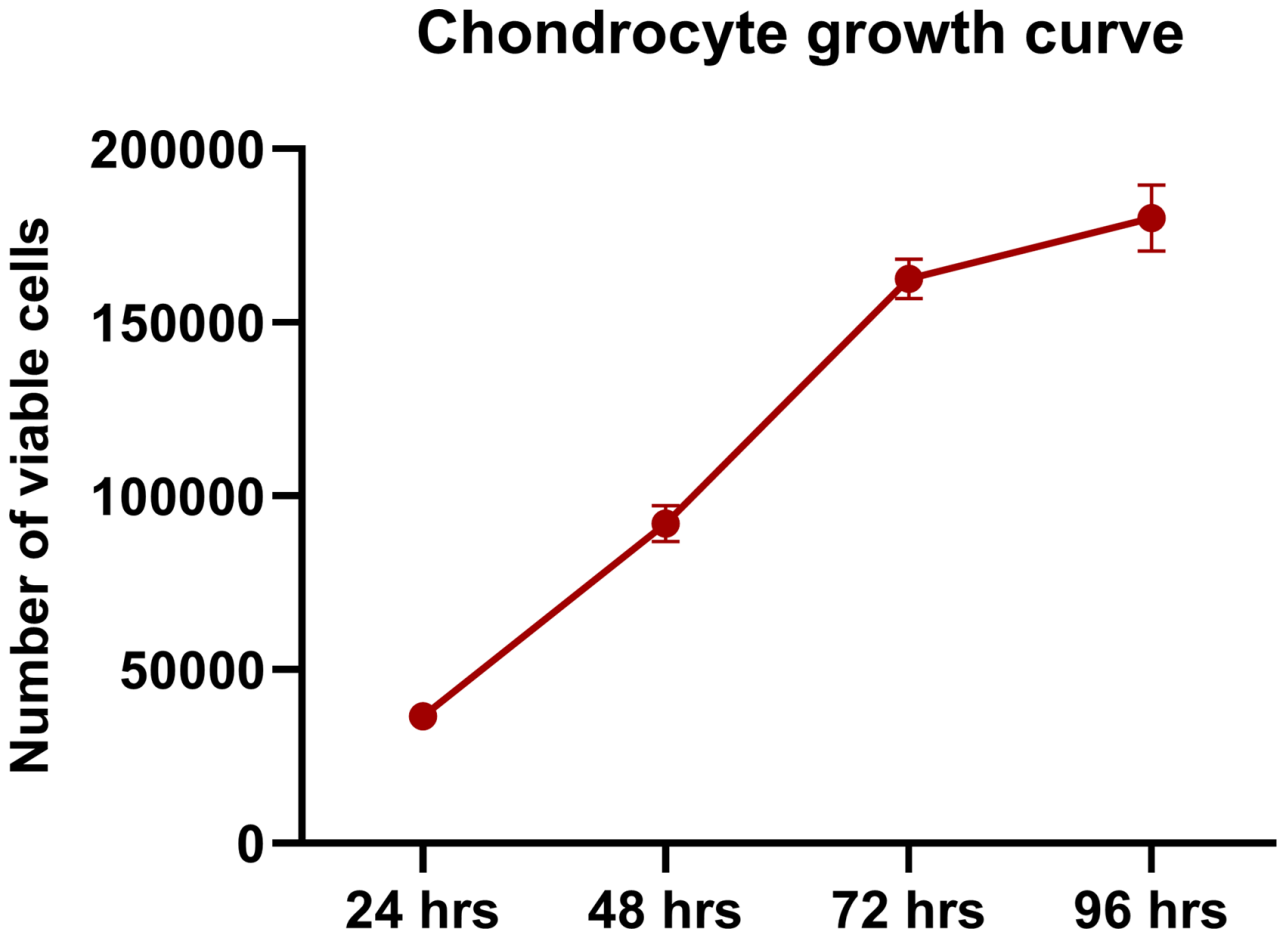
Chondrocyte growth curve. Chondrocytes showed rapid proliferation, with a notable increase in cell numbers between 24 and 72 h. By 96 h, the proliferation rate slowed due to space constraints, indicating an optimal growth environment during the early phase. Data are presented as mean ± SEM.

Growth assessment showed a high proliferation phase starting at 24 h, with cells rapidly dividing and increasing in number. This rapid growth continued over the next 48–72 h, with the culture reaching 80–90% confluency, nearly covering the entire surface. However, as space became limited, cell division slowed down between 72 and 96 h (Figure 2).

#### 3.1.2 Alcian blue staining for glycosaminoglycans

**Figure 3:**
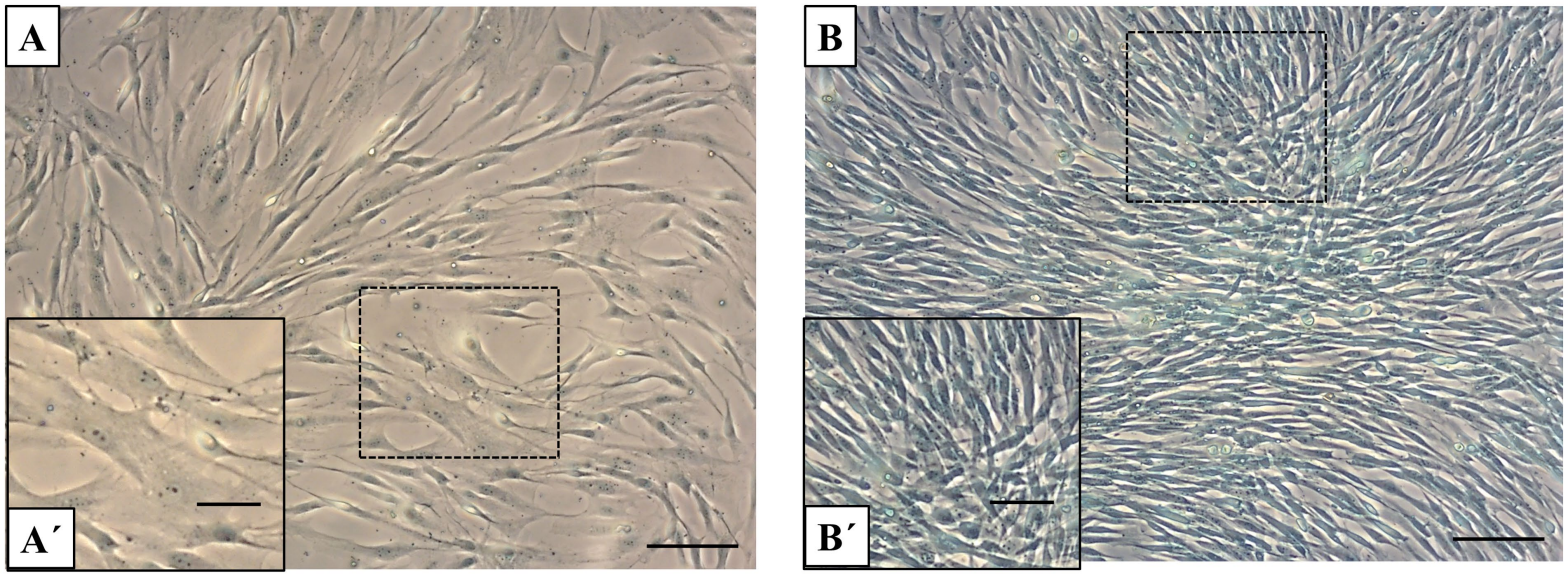
Alcian blue staining in fibroblasts and chondrocytes. **(A)** Alcian blue staining of fibroblast monolayers shows light or minimal staining. **(B)** In contrast, chondrocyte cultures display strong positive alcian blue staining, indicating substantial GAG accumulation after 1 week of culture. Scale bars: **(A,B)** = 50 μm, **(A´,B´)** = 20 μm.

The results of Alcian blue staining revealed a significant difference in GAG accumulation between fibroblast cultures and primary chondrocytes (Figures 3A,B). Chondrocytes, which are responsible for maintaining the extracellular matrix (ECM) of articular cartilage, display strong alcian blue staining (Figure 3B). This indicates that chondrocytes produce a significant amount of GAGs within just 1 week of culture, highlighting their essential role in synthesizing these ECM components. In contrast, primary fibroblast cultures show relatively low staining intensity, suggesting limited GAG synthesis compared to chondrocytes (Figure 3A). The increased staining intensity in chondrocytes underscores their ability to produce and deposit GAGs, which are crucial for cartilage structure and function, even under short-term culture conditions.

#### 3.1.3 Immunocytochemistry of chondrogenic markers

**Figure 4:**
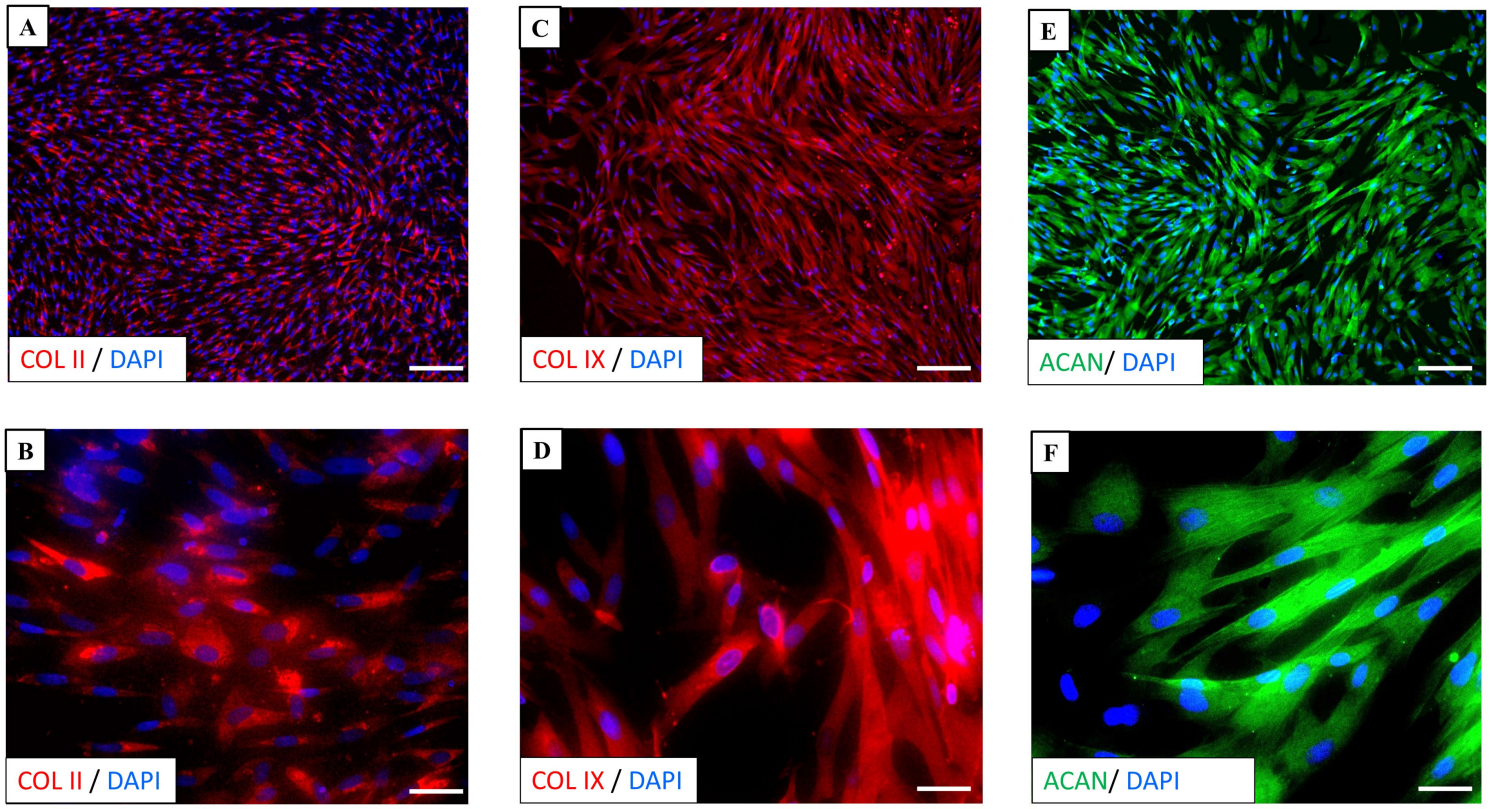
Immunocytochemistry of chondrocyte culture. **(A,B)** Immunostaining for type II collagen (COL II) (red) was primarily localized in the cytoplasm of chondrocytes. **(C,D)** Type IX collagen (COL IX) (red) and **(E,F)** aggrecan (ACAN) (green) staining were observed in the cytoplasm and extended to processes. Nuclei (blue) are counterstained with DAPI. Scale bars: **(A,C,E)** = 50 μm, **(B,D,F)** = 10 μm.

Immunocytochemistry of neonatal chondrocyte cultures was performed in LabTek chamber slides at P1, using typical chondrogenic markers. Immunostaining for type II collagen (COL II) was primarily localized in the cytoplasm of chondrocytes, confirming its active synthesis (Figures 4A,B). Similarly, type IX collagen (COL IX) (Figures 4C,D) and aggrecan (ACAN) (Figures 4E,F) showed positive staining within the cytoplasm, extending into the cellular processes, underscoring their important roles as key components in cartilage. No specific staining for each marker was observed in the negative controls (Supplementary Figure S1).

These findings confirm the presence of all the markers analyzed, indicating that the chondrocytes effectively maintain their chondrogenic phenotype and support the integrity of extracellular matrix.

### 3.2 XTT assay: chondrocyte metabolic activity after exposure to ESM and H_2_O_2_

**Figure 5:**
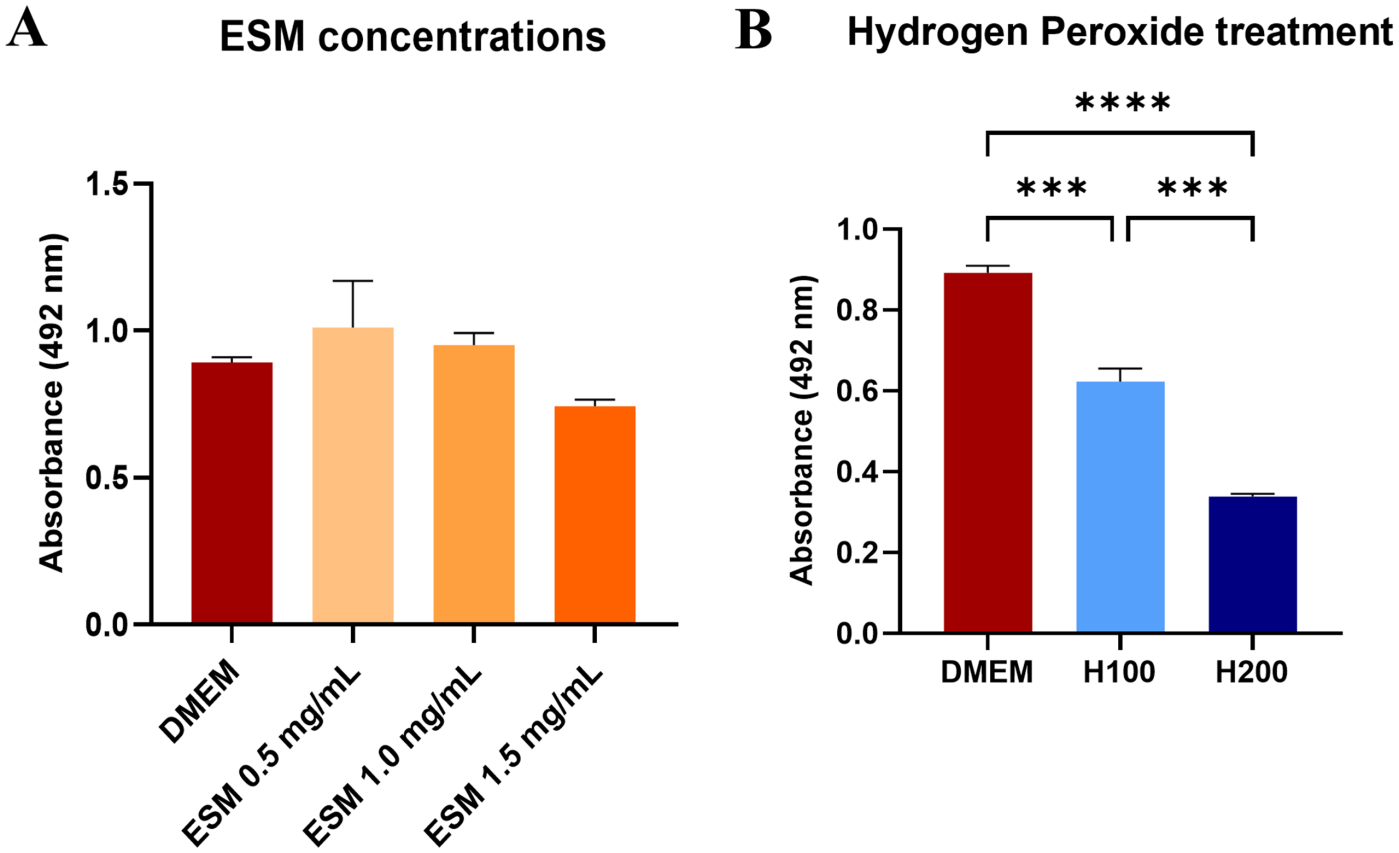
Metabolic activity of chondrocytes exposed to ESM and H_2_O_2_. **(A)** Cell metabolic activity was determined by XTT assay after 24 h of incubation in DMEM (control) and ESM (0.5, 1, 1.5 mg/mL). **(B)** Metabolic activity of chondrocytes after 60 min treatment with 100 μM (H100) and 200 μM (H200) H_2_O_2._ in comparison to DMEM. * *p*  ≤  0.05, ** *p* ≤  0.01, *** *p*  ≤  0.001, **** *p* ≤  0.0001. In all cases GraphPad Prism software was used for statistical analysis, utilizing ANOVA Dunnett’s multiple comparisons test.

To verify that ESM is not cytotoxic and does not negatively impact metabolic activity, primary cultures of canine chondrocytes were cultured in standard DMEM medium and with ESM at concentrations of 0.5, 1, and 1.5 mg/mL. After 24 h, metabolic activity was assessed by standard colorimetric XTT assay (Figure 5A). We did not observe a significant decrease in metabolic activity in any of the experimental ESM groups, however, at concentrations of 0.5 and 1 mg/mL we even observed values higher compared to the DMEM control. Therefore, we have concluded that ESM did not impair metabolic activity. We proceeded by inducing oxidative stress using H_2_O_2_ at two concentrations of 100 μM (H100) and 200 μM (H200), where a significant decrease in metabolic activity was observed at H200 compared to H100 (*** *p* ≤ 0.001) as well as H200, H100 to the DMEM control (**** *p* ≤ 0.0001, *** *p* ≤ 0.001, respectively), represented by chondrocytes cultured in standard DMEM medium (Figure 5B). This demonstrates that while ESM does not impair metabolic activity and might even enhance it, oxidative stress at higher levels (H200) significantly reduces metabolic activity compared to the control.

### 3.3 Cytoprotective effect of ESM on H_2_O_2_ stressed chondrocytes

#### 3.3.1 XTT assay: ESM pre-treatment/post-treatment

**Figure 6:**
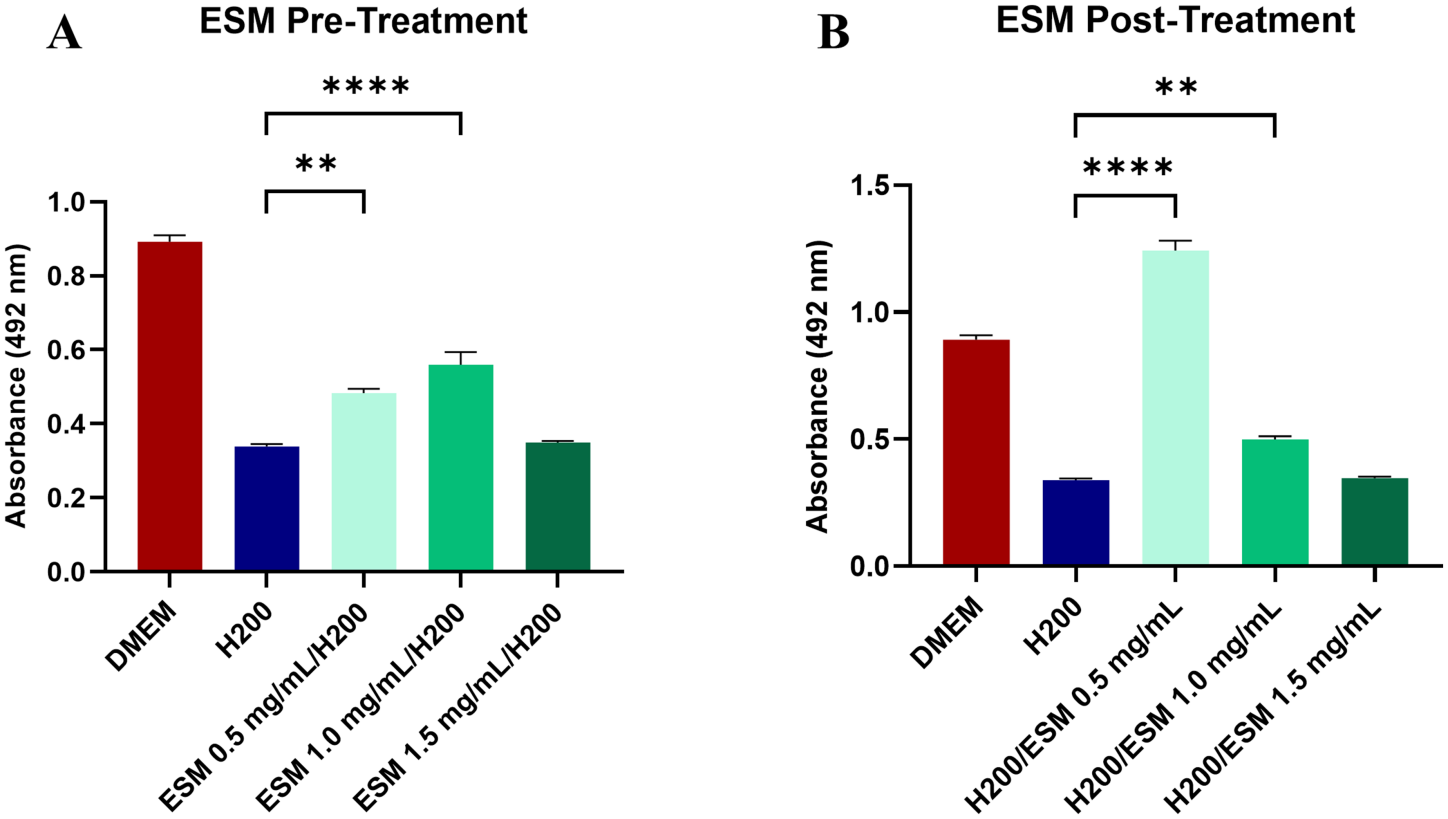
The impact of ESM on chondrocytes under stress. **(A)** XTT assay results of chondrocytes pre-treated with ESM at three concentrations (0.5, 1, 1.5 mg/mL) followed by 60 min culture in 200 μM (H200) H_2_O_2_. **(B)** Data showing metabolic activity in cells treated with H200 followed by the addition of ESM. GraphPad Prism software was used for statistical analysis, ANOVA Dunnett’s multiple comparisons test (** *p* ≤ 0.01, **** *p* ≤  0.0001).

To analyze the functional impact of ESM on the H_2_O_2_-induced oxidative stress model, we therefore treated the chondrocytes with H_2_O_2_. Our study continued by obtaining data on whether pre-treatment or post-treatment of chondrocytes with ESM in three concentrations of 0.5, 1, and 1.5 mg/mL can modulate oxidative stress and reduce metabolic activity induced by H_2_O_2_. We have assessed whether ESM helps to prevent oxidative damage (in pre-treatment) or aids in repairing or minimizing the effects of oxidative damage after it occurs (in post-treatment). In the pre-treatment, we proceeded by culturing canine chondrocytes for 24 h in ESM and after washing in DPBS, the cells were exposed to H200 for 60 min. The functional effect on metabolic activity was evaluated with the XTT test. Our results showed a significantly increased metabolic activity compared to cells cultured only in DMEM and treated with H200 in both ESM 0.5 (** *p* ≤ 0.01) and ESM 1 groups (**** *p* ≤ 0.0001). At the same time, ESM 1 proved to be the group with the most positive pre-treatment effect (Figure 6A). We also evaluated the positive effect of ESM in the case of post-treatment, where we first added H200 to the cells for 60 min, and after washing in DPBS, we analyzed whether ESM can positively affect metabolic activity reduced by hydrogen peroxide. The data from the XTT test showed that the ESM 0.5 (**** *p* ≤ 0.0001) and the ESM 1 (** *p* ≤ 0.01) significantly increased metabolic activity in chondrocytes compared to cells cultured in DMEM after H200 treatment. Overall, these results suggest a cytoprotective potential of ESM against hydrogen peroxide-induced oxidative stress (Figure 6B).

### 3.4 Assessing ESM cytotoxicity on primary chondrocytes using RTCA

**Figure 7:**
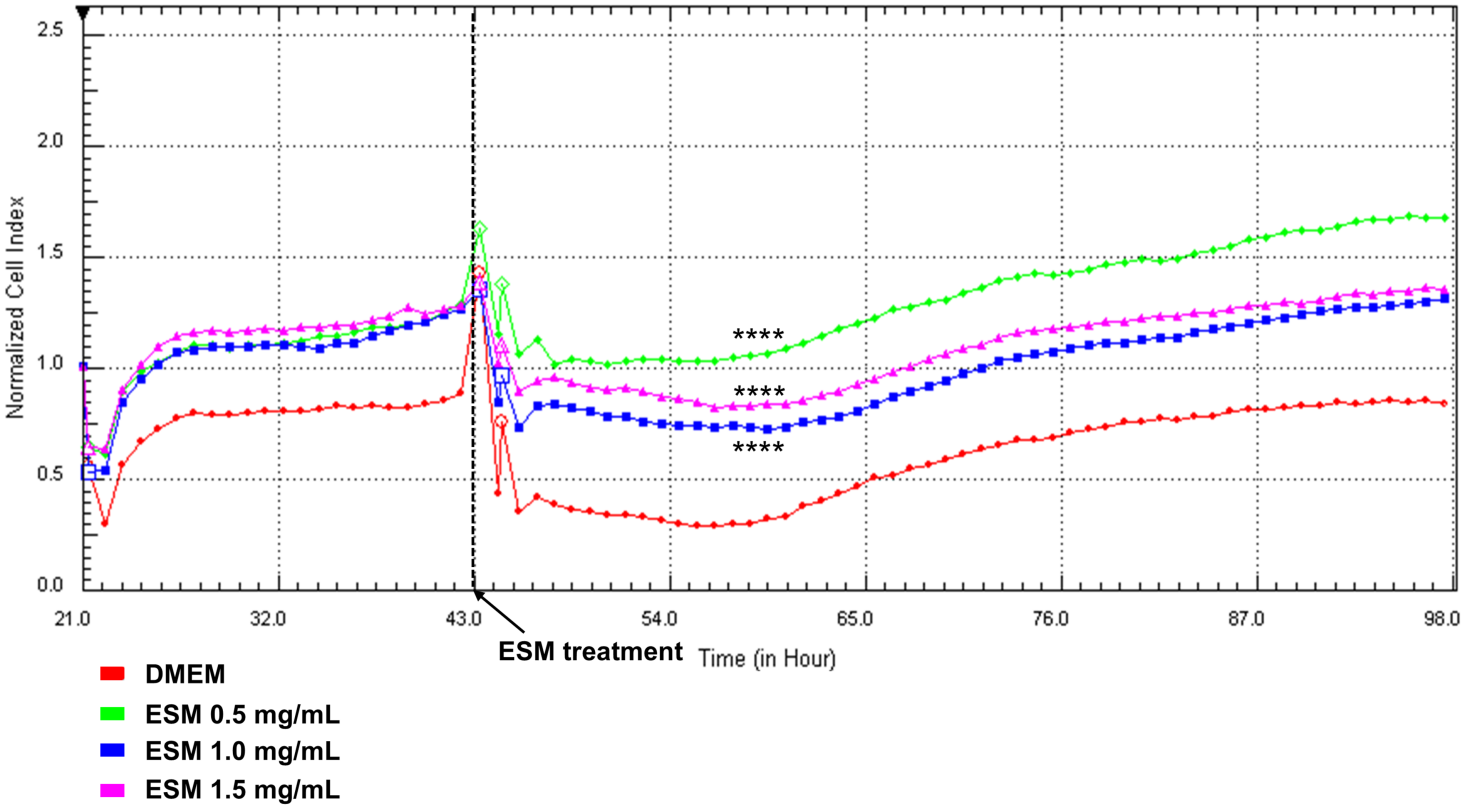
RTCA analysis of chondrocytes exposed to ESM. Chondrocytes were cultured in DMEM and after 48 h in different concentrations of ESM [0.5 mg/mL; 1 mg/mL; 1.5 mg/mL] and DMEM (Control) for 98 h. Concentrations of ESM 0.5 and 1 mg/mL induced a significant increase in CI compared to control. Results are presented as a means; Kruskal-Wallis test was performed **** *p* ≤ 0.0001.

Using Real-Time Cell Analysis (RTCA), we evaluated the cytotoxicity of ESM on primary chondrocytes in real-time. Consistent with the XTT assay, we assessed three concentrations of ESM (0.5, 1, and 1.5 mg/mL). The data collected for 98 h showed that ESM concentrations of 0.5, 1 and 1.5 mg/mL (**** *p* ≤ 0.0001) significantly increased the CI (representing cell adhesion and proliferation) compared to the DMEM (Figure 7).

### 3.5 Assessing ESM pre-treatment and post-treatment effects on H_2_O_2_-stressed chondrocytes using RTCA

#### 3.5.1 Pre-treatment

**Figure 8:**
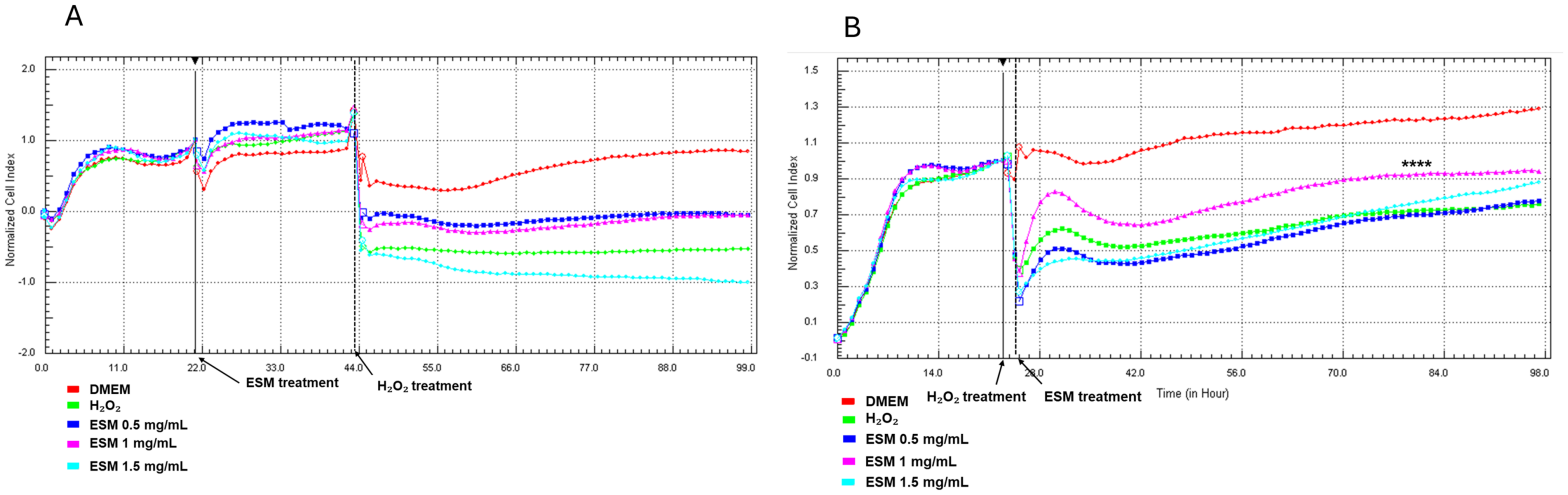
RTCA analysis of ESM pre- and post-treatment effects on stressed chondrocytes. Time-dependent adherence profile of chondrocytes in response to **(A)** 24 h ESM pre-treatment (0.5, 1, 1.5 mg/mL), 1 h incubation with H200, followed by culture in DMEM for 98 h; **(B)** 1 h cultivation of cells in H200, followed by post-treatment with ESM (0.5, 1, 1.5 mg/mL) for 98 h. Statistical significance was evaluated using Kruskal-Wallis test (**** *p* ≤ 0.0001).

Neonatal chondrocytes were cultured in DMEM or ESM (0.5, 1, 1.5 mg/mL) for 24 h. After washing, the cells were exposed to H₂O₂ (H200) for 1 h, followed by the addition of DMEM to all wells. The RTCA results showed that chondrocytes pretreated with ESM (0.5, 1, 1.5 mg/mL) or DMEM and subsequently exposed to H₂O₂ (H200) exhibited a decreased cell index (CI), dropping below 0. This indicates that the cells were undergoing significant loss of adhesion and viability, suggesting progressive cell death (Figure 8A). The DMEM group, which consisted of cells not exposed to peroxide, demonstrated survival with a cell index (CI) reaching up to 1, indicating maintained adhesion and viability under these conditions.

#### 3.5.2 Post-treatment

Following the initial 24 h cell seeding, chondrocytes were exposed to H200 for 1 h. After washing, the cells were cultured in either DMEM or ESM (0.5, 1, 1.5 mg/mL). As illustrated in Figure 8B, the group treated with 1 mg/mL ESM (ESM 1, **** *p* ≤ 0.0001) demonstrated significantly improved cell adhesion compared to the H200 group, where cells exposed to H200 were cultured in DMEM. This enhancement in adhesion was consistently observed during the 25 to 98 h analysis period. To summarize the CI values at 24, 48, and 72 h across all experimental conditions, Supplementary Table S1 was included, showing a comparative key trends and variations observed during the experiment.

The value of the Pearson correlation coefficient between the results of the XTT test and the RTCA 0,4,758 which indicates a moderate positive linear correlation. Collectively, the obtained data indicate a positive functional effect on the improvement of oxidative stress induced by H_2_O_2_ also in the context of adherence and proliferation.

## 4 Discussion

Eggshell membrane (ESM) is a widely available by-product of the chicken egg processing industry, generated in large quantities during the breaking of eggs. This thin membrane, located between the eggshell and the egg white, plays a crucial role in protecting the egg contents and providing structural support during the mineralization process of the eggshell (20). Given its rich composition of chondroprotective proteins and bioactive compounds, ESM holds significant promise as a low-cost ingredient for therapeutic scaffolds in regenerative medicine (21). Its unique properties could be harnessed to develop innovative solutions for cartilage repair and other tissue engineering applications, making it a valuable resource in both veterinary and human medicine (22). Despite this, its use in the prevention and treatment of cartilage damage in veterinary medicine remains underexplored. In this study, we investigated the impact of oxidative stress on chondrocytes, which contributes to chronic inflammation and often leads to irreversible cartilage degeneration in osteoarthritis (23). Although cartilage degeneration is more common in older animals, neonatal chondrocytes are particularly useful in studying cartilage regeneration due to their high proliferative capacity and stable chondrogenic phenotype. These properties make neonatal chondrocytes an ideal model for evaluating the protective and reparative effects of ESM. Moreover, the insights gained from these models can be applied to develop strategies for cartilage repair in adult animals, as the fundamental regenerative mechanisms often remain consistent across different ages (24, 25).

Although many supplements based on ESM on the market claim to alleviate pain, stiffness, and other osteoarthritis symptoms, there is limited reliable data on their specific mechanisms, particularly in the context of oxidative stress (26).

Therefore, the main objective of our study was to assess the effects of commercially available natural ESM under *in vitro* conditions, utilizing an optimized neonatal chondrocyte model to better understand its potential in mitigating oxidative stress. To achieve this, we isolated and characterized canine neonatal chondrocytes, confirming their identity as committed chondrocytes. These cells demonstrated rapid growth between 24 and 72 h, along with increased production of sulfated proteoglycans, as confirmed by Alcian blue staining. This aligns with previous studies on immature murine articular chondrocytes, which showed similar growth patterns and proteoglycan production (27). The typical chondrogenic phenotype was further validated by the high expression of Collagen II, IX, and aggrecan—similar to what was observed in human articular chondrocyte cultures derived from infant, juvenile, and adult sources (28). However, some differences in chondrogenic properties were noted, with adult chondrocytes showing signs of partial dedifferentiation after a longer subculture, unlike juvenile chondrocytes (24). These data align with our findings, revealing the stable chondrogenic capacity of canine neonatal chondrocytes, making them well-suited for our *in vitro* studies (25, 29). After refining the *in vitro* platform, we evaluated the cytotoxic impact of specific ESM concentrations on neonatal chondrocytes. Our results showed that ESM, especially at doses 0.5 and 1 mg/mL, showed greater or lesser protective effects in different setups. Based on previous findings, the beneficial effects of ESM can be attributed, in part, to its bioactive components, including collagen, glycosaminoglycans, hyaluronic acid, and growth factors. These elements work together to enhance cellular energy metabolism by promoting mitochondrial activity and improving nutrient absorption (3). Particularly, glycosaminoglycans play a crucial role in regulating cell signalling, influencing both growth and metabolism (30). They may act as coreceptors between cells and growth factors, such as Vascular Endothelial Growth Factor (VEGF), controlling metabolic and proliferative processes (31). Hyaluronic acid supports metabolic activity by facilitating nutrient exchange and binding directly to cell surface receptors like CD44, potentially enhancing cell signalling pathways involved in growth and repair (32). Transforming Growth Factor-*β* (TGF-β) and Fibroblast Growth Factor (FGF), both found in ESM, are important trophic factors that promote chondrocyte proliferation, matrix synthesis, and differentiation (33). In addition, TGF-β is a key regulator of chondrocyte viability and proliferation, promoting cell growth, enhancing extracellular matrix production, inhibiting apoptosis, and maintaining the chondrogenic phenotype (34). FGF stimulates the proliferation of chondrocytes by activating specific signalling pathways, such as the MAPK/ERK pathway, which leads to increased expression of genes associated with the cell cycle, promoting cell division and increasing the number of viable chondrocytes (35). These results enabled us to investigate ESM’s dual role in preventing and treating metabolic impairment in canine neonatal chondrocytes exposed to oxidative stress. In the XTT metabolic assay, we observed that ESM concentrations of 0.5 and 1 mg/mL partially protected against H₂O₂ effects in pre- and post-treatment setups. However, higher concentrations did not show additional protection. This could suggest that 0.5 and 1 mg/mL represent an optimal threshold for ESM’s protective action, while higher concentrations may not further enhance the cellular response and might even lead to saturation or reduced efficacy. Furthermore, primary cultures of neonatal chondrocytes could be more sensitive to higher concentrations of ESM. Their proliferative capacity and metabolic activity are highly responsive to environmental changes, and excessive concentration may lead to cellular stress or toxicity. This aligns partially with our initial analysis, where we compared the effects of all three ESM concentrations (0.5, 1, and 1.5 mg/mL) with DMEM. The data revealed that 0.5 and 1 mg/mL were the optimal concentrations for chondrocyte metabolic activity, while 1.5 mg/mL did not show additional benefits. This suggests that neonatal chondrocytes may have a limited capacity to handle higher concentrations, supporting the hypothesis that higher doses could lead to reduced efficacy or potential cellular stress.

Data from the RTCA assay indicated that ESM at 1 mg/mL only in the post-treatment setup partially restored cell adhesion, suggesting some recovery after oxidative stress. In contrast, pre-treatment with all tested concentrations showed no significant effect, implying that pre-treatment may not offer adequate protection against oxidative stress. This discrepancy between the XTT and RTCA findings emphasizes the context-dependent impacts of ESM preconditioning on chondrocytes. The XTT assay showed increased metabolic activity after ESM preconditioning, indicating enhanced mitochondrial function. This suggests that ESM may improve the metabolic resilience of chondrocytes under oxidative stress. On the other hand, RTCA monitors real-time changes in cell adhesion, proliferation, and morphology (36). The lack of significant effects in RTCA suggests that ESM preconditioning may not improve chondrocytes’ structural or adhesive properties under oxidative stress. In conclusion, ESM preconditioning may enhance metabolic activity, but it does not provide adequate protection against severe oxidative damage that impacts cell survival and adhesion (36). Our data are the first suggesting the therapeutic effects of ESM, which may treat already impaired chondrocytes by activating protective pathways. There can be several antioxidant mechanisms and pathways involved. Superoxide Dismutase (SOD), an enzyme found in ESM, may play a direct role in scavenging superoxide radicals, by converting them into less harmful molecules. In H_2_O_2_-induced human SW1353 chondrocytes, hatched ESM significantly increased the cell viability and ameliorated oxidative stress and cartilage matrix degradation by reducing the level of ROS, matrix metalloproteases 3 and 13 and by promoting the expression of SOD and type II collagen. This may be achieved by activating the cellular Keap1/Nrf2/HO-1 pathway. Thus, ESM’s ability to increase SOD activity together with other factors may prevent oxidative damage in cartilage cells (37). Furthermore, ESM may enhance glutathione levels or the activity of Glutathione peroxidase, contributing to the cell’s ability to neutralize oxidative damage and may stimulate catalase activity that breaks down hydrogen peroxide into water and oxygen (38). By supporting mitochondrial health, ESM can help reduce the production of ROS within mitochondria, preventing the excessive accumulation of ROS, and contributing to the overall antioxidant defense of cells (39). Finally, ESM may stimulate autophagy, and inhibit the NF-κB pathway, all contributing to enhanced protection against oxidative stress and support of chondrocyte viability and proteins (40–42).

## 5 Conclusion

The eggshell membrane (ESM) is a significant source of bioactive compounds, including proteins and antioxidants, which are suggested to provide therapeutic benefits by neutralizing reactive oxygen species (ROS) and modulating inflammation associated with chondrocyte damage in osteoarthritis. This study revealed that isolated canine chondrocytes exhibited robust proliferation and enhanced GAG production while maintaining essential cartilage markers. This made them a suitable model for evaluating the effects of ESM proteins on H₂O₂-induced oxidative stress in neonatal canine chondrocytes. Post-treatment with ESM at concentrations of 0.5 and 1 mg/mL improved chondrocyte metabolic activity and proliferation, highlighting ESM’s potential for repair of oxidative damage. Both XTT and RTCA confirmed the results, but the prophylactic effect of ESM (at the same concentrations) was only observed through XTT. These assays reveal different aspects of cellular health, with XTT sensitive to metabolic changes under oxidative stress, while RTCA might not capture structural resilience or adhesion under similar conditions. Our findings particularly emphasize the therapeutic potential of ESM proteins in supporting cartilage health. Furthermore, we have developed a reliable platform to assess dietary supplements designed to prevent or repair cartilage damage in dogs. This research provides valuable insights into the practical applications of ESM proteins in veterinary medicine.

## Data Availability

The raw data supporting the conclusions of this article will be made available by the authors, without undue reservation.

## Supplementary material

SUPPLEMENTARY FIGURE S1 Immunocytochemical analysis of negative controls in chondrocyte cultures. No specific staining was detected for any marker in the negative controls, including type II collagen (-COL II) (red) **(A)**, type IX collagen (-COL IX) (red) **(B)**, and aggrecan (-ACAN) (green) **(C)**. Nuclei were counterstained with DAPI (blue). Scale bars: **(A–C)** = 50 μm.
