# Supplementary figures and images for: Impact of eggshell membrane on metabolism and cell adhesion in oxidatively stressed canine chondrocytes
Source: Front Vet Sci. 2025 Jan 8;11:1517349. doi: 10.3389/fvets.2024.1517349 (PMC11751048; doi:10.3389/fvets.2024.1517349)

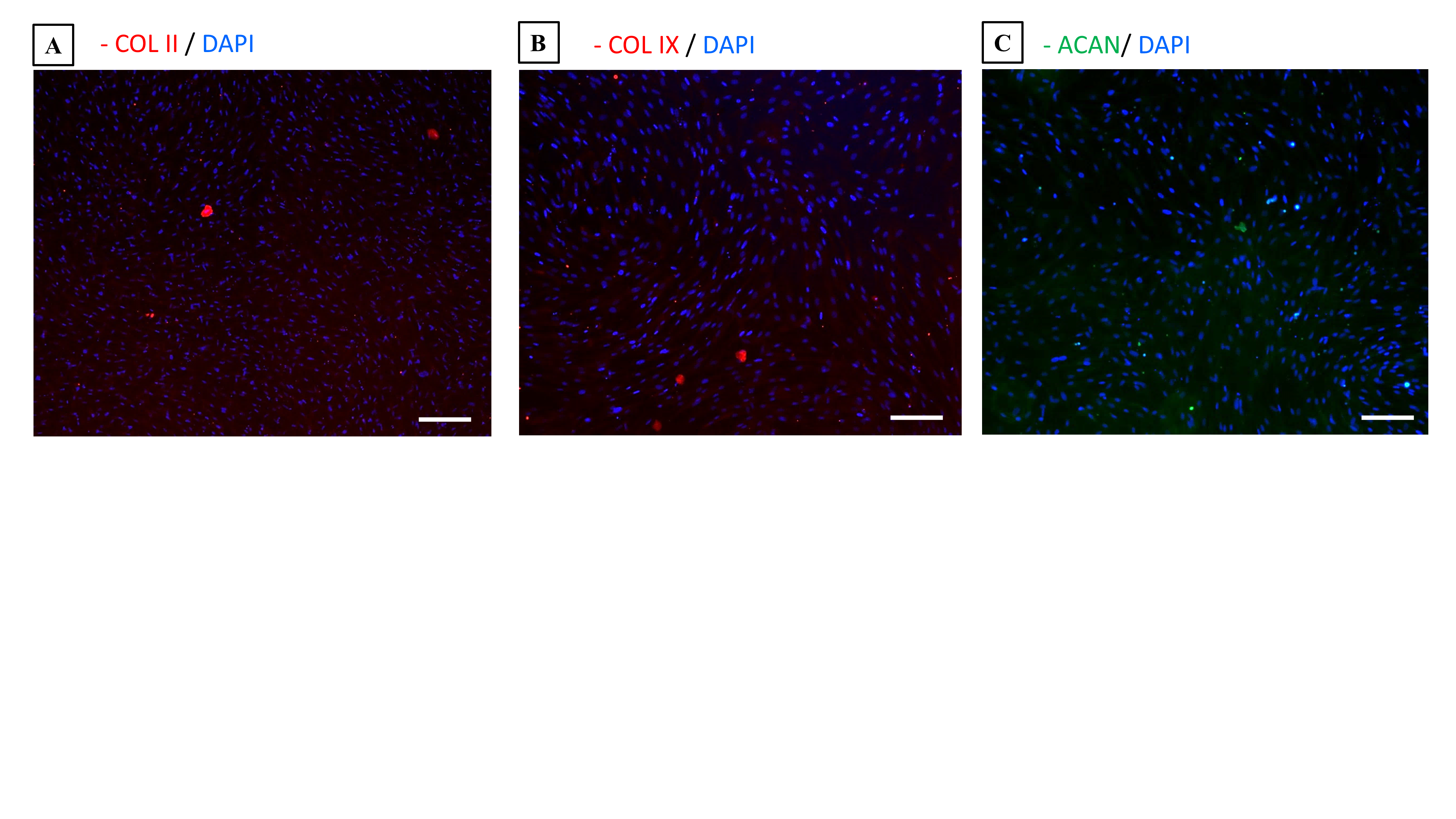

Supplement: SUPPLEMENTARY FIGURE S1 — Immunocytochemical analysis of negative controls in chondrocyte cultures. No specific staining was detected for any marker in the negative controls, including type II collagen (-COL II) (red) (A), type IX collagen (-COL IX) (red) (B), and aggrecan (-ACAN) (green) (C). Nuclei were counterstained with DAPI (blue). Scale bars: (A–C) = 50 μm. [file Image_1.TIF]
