# Supplementary material for: Impact of eggshell membrane on metabolism and cell adhesion in oxidatively stressed canine chondrocytes
Source: Front Vet Sci. 2025 Jan 8;11:1517349. doi: 10.3389/fvets.2024.1517349 (PMC11751048; doi:10.3389/fvets.2024.1517349)
Supplement: Supplementary file 2 [file Table_1.DOCX]

| **% adherence decrease ± SD** | | | |
| --- | --- | --- | --- |
|  | **24 h ± SD** | **48 h ± SD** | **72 h ± SD** |
| **Treatment** | | | |
| **DMEM** | 100.00 ± 34.09 | 100.00 ± 33.49 | 100.00 ± 26.78 |
| **E0.5** | 158.83 ± 27.56 | 126.40 ± 31.08 | 159.82 ± 34.12 |
| **E1.0** | 111.12 ± 11.14 | 100.61 ± 7.43 | 129.76 ± 3.84 |
| **E1.5** | 127.12 ± 37.99 | 105.46 ± 30.83 | 128.34 ± 59.56 |
| **Pre-treatment** | | | |
| **DMEM** | 100.00±34.09 | 100.00±33.49 | 100.00 ± 26.78 |
| **H200** | – | – | – |
| **E0.5** | – | – | – |
| **E1** | – | – | – |
| **E1.5** | – | – | – |
| **Post-treatment** | | | |
| **DMEM** | 100.00 ± 12.54 | 100.00 ± 7.07 | 100.00 ± 6.84 |
| **H200** | 50.39 ± 27.44 | 58.42 ± 24.66 | 59.07 ± 18.07 |
| **E0.5** | 42.24 ± 8.13 | 55.02 ± 7.73 | 60.16 ± 9.35 |
| **E1** | 61.70 ± 7.45 | 74.86 ± 14.00 | 73.85 ± 10.09 |
| **E1.5** | 44.62 ± 11.95 | 58.12 ± 14.77 | 67.74 ± 11.37 |

**Table 1**. Summary of CI values at selected time points (24, 48 and 72 hours) across all experimental conditions. Results are expressed in percentage values in comparison to control (100%). Data are presented as mean ± SD. **–** (data out of range).
